# Quantification of Lumefantrine in Human Plasma Using LC-MS/MS and Its Application to a Bioequivalence Study

**DOI:** 10.1155/2013/437697

**Published:** 2012-09-23

**Authors:** Satish G. Pingale, Kiran V. Mangaonkar

**Affiliations:** Analytical Chemistry Research Laboratory, Mithibai College of Arts, Chauhan Institute of Science & Amrutben Jivanlal College of Commerce & Economics, Vile Parle (W), Mumbai 400056, India

## Abstract

An analytical method based on protein precipitation has been developed and validated for analysis of lumefantrine in human plasma. Artesunate was used as an internal standard for lumefantrine. Inertsil ODS column provided chromatographic separation of analytes followed by detection with mass spectrometry. The method involves simple isocratic chromatographic condition and mass spectrometric detection in the positive ionization mode using an API-3000 system. The total run time was 2.5 minutes. The proposed method has been validated with linear range of 200–20000 ng/mL for lumefantrine. The intrarun and interrun precision values are within 6.66% and 5.56%, respectively, for lumefantrine at the lower limit of quantification level. The overall recovery for lumefantrine and artesunate was 93.16% and 91.05%, respectively. This validated method was used successfully for analysis of plasma samples from a bioequivalence study.

## 1. Introduction

Lumefantrine (*IUPAC name: 2-(dibutylamino)-1-[(9Z)-2,7-dichloro-9*-*(4-chlorobenzylidane)-9H-fluoren-4-yl]ethan-1-ol*) is an antimalarial agent used to treat acute uncomplicated malaria. It is administered in combination with artemether for improved efficacy. This combination therapy exerts its effects against the erythrocytic stages of *Plasmodium* and may be used to treat infections caused by *P*. *falciparum* and unidentified plasmodium species, including infections acquired in chloroquine-resistant areas [1].

Few bioanalytical methods are reported to determine lumefantrine in plasma using HPLC-UV [2–4] and LC-MS/MS [5–8] detection. All these reported methods require total run time ranging from 5 to 17 min.

Pharmacokinetic study in five healthy volunteers under fasting condition was studied by Cesar et al. [8]. Bioequivalence study with comparative safety evaluation was conducted on 72 healthy Indian human subjects under a fed condition by Khandave et al. [6].

All reported methods have long run time. Hence, it felt necessary to develop and validate a rapid and selective method that can be successfully applied to a bioequivalence study.

In the present paper we would like to present a simple and high-throughput protein precipitation method for quantification of lumefantrine using artesunate as an internal standard with LC-MS/MS detection. The application of this validated method in analyzing samples from a bioequivalence study involving lumefantrine is also presented.

## 2. Experimental

### 2.1. Chemicals and Reagents

The reference standard of lumefantrine was provided by Ipca Laboratories Ltd. (Mumbai, India). The reference standard artesunate was obtained from Sigma Aldrich (St. Louis, MI, USA). Purity of both the standards was higher than 99%. The lumefantrine tablets, containing 120 mg lumefantrine per tablet, were obtained from Novartis Pharmaceuticals Corp., Suffern, New York. High-purity water was prepared in-house using a Milli-Q A10-gradient water purification system (Millipore, Bangalore, India). LC-grade methanol and acetonitrile were purchased from J.T. Baker Inc. (Phillipsburg, NJ, USA). AR-grade formic acid and hydrochloric acid were procured from Merck (Mumbai, India). Drug-free (blank) human plasma containing heparin was obtained by enrolling healthy volunteers and taking their consent before bleeding. The plasma thus obtained was stored at −20°C prior to use.

### 2.2. Calibration Curve and Quality Control Samples

Two separate stock solutions of lumefantrine were prepared for bulk spiking of calibration curve and quality control samples for the method validation exercise as well as the subject sample analysis. The stock solutions of lumefantrine and artesunate were prepared in methanol : acetonitrile : 0.1 N hydrochloric acid (70 : 30 : 0.05 v/v) at free base concentration of 2500 *μ*g/mL. Primary dilutions and working standard solutions were prepared from stock solutions using water : methanol (30 : 70 v/v) solvent mixture. These working standard solutions were used to prepare the calibration curve and quality control samples. Blank human plasma was screened prior to spiking to ensure that it was free of endogenous interference at retention times of lumefantrine and internal standard artesunate. An eight-point standard curve and four quality control samples were prepared by spiking the blank plasma with an appropriate amount of lumefantrine. Calibration samples were made at concentrations of 200, 400, 2000, 4000, 8000, 12000, 16000, and 20000 ng/mL, and quality control samples were made at concentrations of 200, 600, 10000, 17000, and 20000 ng/mL for lumefantrine.

### 2.3. Liquid Chromatography and Mass Spectrometric Conditions

**Figure 1:**
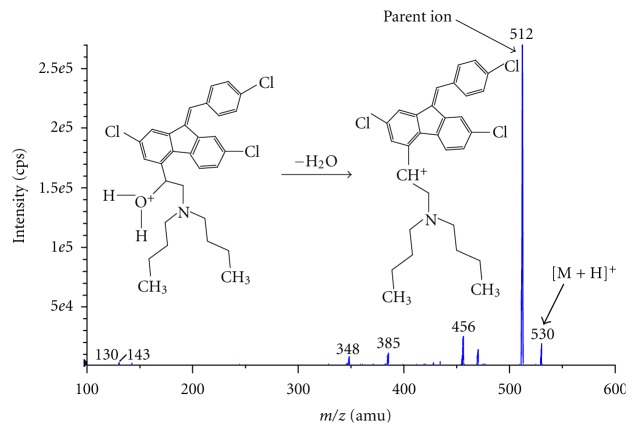
Product ion mass spectrum of lumefantrine.

**Figure 2:**
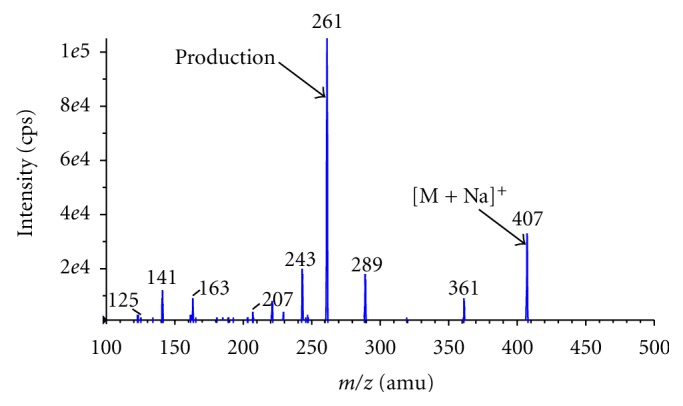
Product ion mass spectrum of artesunate.

Chromatographic separation was carried out on a Shimadzu LC (Kyoto, Japan) with a Inertsil ODS-2V column (50 × 4.6 mm, 5 *μ*m) purchased from GL Sciences Inc., Japan. A mobile phase consisting of methanol, acetonitrile, and 0.1% formic acid in water solution in the ratio of 56 : 24 : 20 v/v/v was delivered with a splitter at a flow rate of 1 mL/min. The total run time for each sample analysis was 2.5 minutes. Mass spectra were obtained using an API-3000 from Applied Biosystems, Canada, equipped with electrospray ionization source. The mass spectrometer was operated in the multiple reaction monitoring (MRM) mode. Electrospray ionization in the positive ion mode was used for sample introduction and ionization. Source-dependent parameters optimized were as follows: nebulizer gas flow: 8 L/min; auxiliary gas flow: 8 L/min; ion spray voltage (ISV): 5500 V, and temperature (TEM): 400°C. The compound-dependent parameters such as the declustering potential (DP), focusing potential (FP), entrance potential (EP), collision energy (CE), cell exit potential (CXP) were optimized during tuning as 100, 360, 10, 41, 10 and 30, 110, 10, 24, 10 eV for lumefantrine and artesunate, respectively. The collision-activated dissociation (CAD) gas was set at 4 psi, while the curtain gas flow was set at 6 L/min using nitrogen gas. Quadrupole 1 and quadrupole 3 were both maintained at unit resolution and dwell time was set at 200 ms for lumefantrine and artesunate. The mass transitions were selected as *m/z* 530 → 512 for lumefantrine and *m/z* 407 → 261 for artesunate. The product ion mass spectra for lumefantrine and artesunate are represented in Figures 1 and 2, respectively. 

The data acquisition was ascertained by Analyst 1.4.2 software. For quantification, the peak area ratios of the target ions of the analyte to those of the internal standard were compared with weighted (1/*x* ^2^) least squares calibration curves in which the peak area ratios of the calibration standards were plotted versus their concentrations.

### 2.4. Plasma Sample Preparation

An internal standard working solution (250 *μ*g/mL of artesunate) of 10 *μ*L was spiked in 0.1 mL aliquot of human plasma sample. To this 1.8 mL of mobile phase was added and vortexed for 3 minutes. The samples were then centrifuged for 10 minutes at 15000 rpm and 5 *μ*L of supernatant layer was injected into the LC-MS/MS system through the autosampler. 

### 2.5. Validation

A thorough and complete method validation of lumefantrine in human plasma was carried out following US FDA guidelines [9]. The method was validated for selectivity, sensitivity, matrix effect, linearity, precision and accuracy, recovery, dilution integrity, partial volume, reinjection reproducibility, and stability. Selectivity was performed by analyzing the human blank plasma samples from six different sources (or donors) with an additional haemolysed group and lipemic group to test for interference at the retention times of analytes. The assessment of matrix effect (coeluting, undetected endogenous matrix compounds that may influence the analyte ionization) constitutes an important and integral part of validation for quantitative LC-MS/MS method for supporting pharmacokinetics studies. It was performed by processing six different lots of plasma samples in quadruplet (*n* = 4). LQC and HQC working solutions were spiked following extraction in duplicate for each lot. The % CV at each level was calculated by taking the mean value obtained by injecting the postextracted samples prepared in duplicate from each plasma lot, which should be less than ten.

The intra-run (within a day, *n* = 3) and inter-run (between days, *n* = 3) accuracy was determined by replicate analysis of quality control samples (*n* = 6) at LLOQ (lower limit of quantification), LQC (low quality control), MQC (medium quality control), HQC (high quality control), and ULOQ (upper limit of quantification) levels. The % CV should be less than 15% and accuracy (% RE) should be within 15% except LLOQ where it should be within 20%.

Accuracy is defined as the percent relative error (% RE) and was calculated using the formula % RE = ((*E *−*T*)/*T*) × 100, where *E* is the experimentally determined concentration and *T* is the theoretical concentration. Assay precision was calculated by using the formula % CV = (SD/*M*) (100), where *M* is the mean of the experimentally determined concentrations and SD is the standard deviation of *M*. The % change was calculated by using the formula % change = (*S*/*F *− 1) × 100, where *S* is the mean concentration of stability samples and *F* is the mean concentration of freshly prepared samples.

The extraction efficiencies of lumefantrine and artesunate were determined by analysis of six replicates at each quality control concentration level for lumefantrine and at one concentration for the internal standard artesunate. The percent recovery was evaluated by comparing the peak areas of extracted standards to the peak areas of unextracted standards (spiked into extracted matrix of same lot).

The dilution integrity experiment was performed with an aim to validate the dilution test to be carried out on higher analyte concentrations above upper limit of quantification (ULOQ), which may be encountered during real subject sample analysis. Dilution integrity experiment was carried out at 1.7 times the ULOQ concentration. Six replicates each of 1/2 and 1/4 concentrations were prepared and their concentrations were calculated by applying the dilution factor 2 and 4 against the freshly prepared calibration curve.

In real subject samples with insufficient plasma volume, the partial volume experiment was performed on medium quality control (MQC) concentration level to validate the method. Six replicates each of half and quarter volume of the total volume of plasma required for processing were prepared and their concentrations were calculated by applying the concentration factor 2 and 4 against the freshly prepared calibration curve.

LQC and HQC samples were injected to check re-injection reproducibility, after which the system was turned off and then restarted after two hours. The same samples were then reinjected, and original values were compared with re-injected values with respect to % change, which should be less than 10%.

As a part of the method validation, stability was evaluated in stock solutions and in plasma under different conditions, maintaining the same conditions that occurred during study samples handling and analysis. Stock solution stability was performed by comparing area response of the analyte and the internal standard in the stability sample, with the area response of sample prepared from fresh stock solution. Stability studies in plasma were performed at LQC and HQC concentration level using six replicates at each level. Analyte was considered stable if the % change is less than 15% as per US FDA guidelines [9]. The stability of the spiked human plasma samples stored at room temperature (bench top stability) was evaluated for 17 h. The stability of the spiked human plasma samples stored at −70°C in coolant (coolant stability) was evaluated for 26 h. The autosampler sample stability was evaluated by comparing the extracted plasma samples that were injected immediately (time 0 h), with the samples that were re-injected after storing in the autosampler at 10°C for 34 h. The reinjection reproducibility was evaluated by comparing the extracted plasma samples that were injected immediately (time 0 h), with the samples that were re-injected after storing in the refrigerator at 2–8°C for 29 h. The freeze-thaw stability was conducted by comparing the stability samples that had been frozen at −70°C and thawed three times, with freshly spiked quality control samples. Six aliquots each of LQC and HQC concentration levels were used for the freeze-thaw stability evaluation. For long-term stability evaluation, freshly prepared calibration curve and quality control samples were injected along with the stability samples. The concentrations obtained after 16, 39, 87, and 221 days intervals were compared with initial concentrations.

### 2.6. Application of Method

The validated method has been successfully used to analyze lumefantrine concentrations in sixty human volunteers under fasting conditions after administration of a single tablet containing 120 mg lumefantrine as an oral dose. The study design was a randomized, two-period, two-sequence, two-treatment single-dose, open-label, bioequivalence study using COARTEM manufactured by Novartis Pharmaceuticals Corp., Suffern, New York, as the reference formulation. The study was conducted according to current GCP guidelines and after signed consent of the volunteers. Before conducting the study, it was also approved by an authorized ethics committee.

There were a total of 25 blood collection time-points including the predose sample, per period. The blood samples were collected in separate vacutainers containing heparin as anticoagulant. The plasma from these samples was separated by centrifugation at 3500 rpm within the range of 2–8°C. The plasma samples thus obtained were stored at −70°C till analysis. Following analysis the pharmacokinetic parameters were computed using WinNonlin software version 5.2 and 90% confidence interval was computed using SAS software version 9.2.

## 3. Results and Discussion

### 3.1. Method Development

During method development different options were evaluated to optimize detection parameters, chromatography, and sample extraction.

#### 3.1.1. Mass Spectra

Electrospray ionization (ESI) provided maximum response over atmospheric pressure chemical ionization (APCI) mode and was chosen for this method. The instrument was optimized to obtain sensitivity and signal stability during infusion of the analyte in the continuous flow of mobile phase to electrospray ion source operated at both polarities at a flow rate of 10 *μ*L/min. Lumefantrine gave more response in positive ion mode as compared to the negative ion mode. The predominant peaks in the primary ESI spectra of lumefantrine and artesunate correspond to the [M + H]^+^ ions at *m/z* 530 and [M + Na]^+^ ions at 407, respectively. 

Major product ions of lumefantrine and artesunate scanned in quadrupole 3 after a collision with nitrogen in quadrupole 2 had an *m/z* of 512 and 261, respectively. 

#### 3.1.2. Chromatography

Initially, a mobile phase consisting of ammonium acetate and acetonitrile in varying combinations was tried, but a low response was observed. The mobile phase containing acetic acid : acetonitrile (20 : 80 v/v) and acetic acid : methanol (20 : 80 v/v) gives better response, but poor peak shape was observed. A mobile phase of 0.1% formic acid in water in combination with methanol and acetonitrile with varying combinations is tried. The best signal along with a marked improvement in the peak shape was observed for lumefantrine and artesunate by using a mobile phase containing 0.1% formic acid in water in combination with methanol and acetonitrile (20 : 56 : 24 v/v/v). 

Short-length columns, such as Symmetry Shield RP18 (50 × 2.1 mm, 3.5 *μ*m), Inertsil ODS-2V (50 × 4.6 mm, 5 *μ*m), HyPURITY C18 (50 × 4.6 mm, 5 *μ*m), and HyPURITY Advance (50 × 4.0 mm, 5 *μ*m) were tried during the method development. Symmetry Shield RP18 column gave a relatively good peak shape but the response was low. Using HyPURITY C18 column poor chromatography was observed. The best signal was obtained using the Inertsil ODS-2V (50 × 4.6 mm, 5 *μ*m) column. It gave satisfactory peak shapes for both lumefantrine and artesunate, and a flow rate of 1 mL/min reduced the run time to 2.5 min. Introducing such a high flow directly into the ionization source affects evaporation of solvents, which further causes improper ionization and reduces response, so a splitter was utilized to control direct flow in the ionization source. The column oven was kept at a constant temperature of about 25°C.

#### 3.1.3. Extraction

Several organic solvents were employed to extract analytes from the plasma sample. All the tested solvents (ethyl acetate, chloroform, hexane, dichloromethane, and methyl tertiary butyl ether) in liquid-liquid extraction yield less recovery. Protein precipitation using acetonitrile and mobile phase was also tried. As compared to the acetonitrile, mobile phase yields high recovery. 

It was difficult to find a compound which could ideally mirror the analytes to serve as a good IS. Several compounds were investigated to find a suitable IS, and finally artesunate belonging to a similar class of compounds was found to be most appropriate for the present purpose. There was no significant effect of IS on analyte recovery, sensitivity, or ion suppression. The results of method validation using artesunate as the IS were acceptable in this study based on FDA guidelines. High recovery and selectivity were observed in the protein precipitation method.

These optimized detection parameters, chromatographic conditions, and extraction procedure resulted in reduced analysis time with accurate and precise detection of lumefantrine in human plasma.

### 3.2. Method Validation

#### 3.2.1. Selectivity and Sensitivity

**Figure 3:**
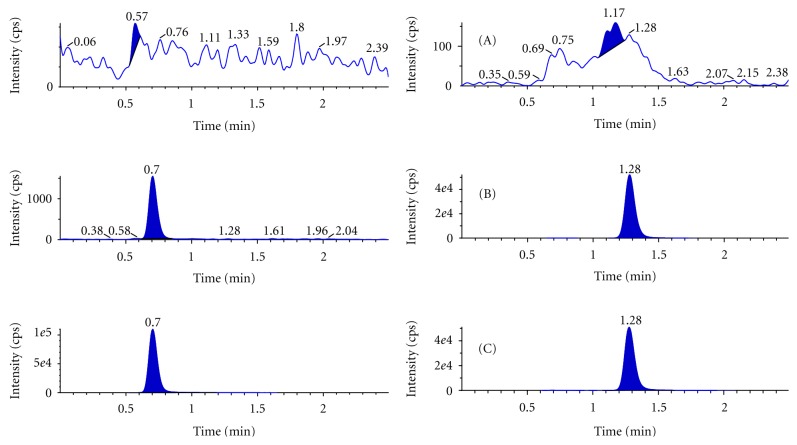
Representative chromatograms of lumefantrine (left) and artesunate (right) in human plasma. (A) Blank plasma, (B) LLOQ, and (C) Real subject sample.

Representative chromatograms obtained from blank plasma, plasma spiked with lower limit of quantification, and real subject sample for lumefantrine and artesunate are shown in Figure 3. The mean % interference observed at the retention time of analytes between eight different lots of human plasma including haemolysed and lipemic plasma containing heparin as an anticoagulant was calculated and the value was found to be 0.00% and 0.00% for lumefantrine and artesunate, respectively, which was within acceptance criteria. Six replicates of extracted samples at the LLOQ level in one of the plasma sample having least interference at the retention time of lumefantrine were prepared and analyzed. The % CV of the area ratios of these six replicates of samples was 3.38% for lumefantrine confirming that interference does not affect the quantification at the LLOQ level. Utilization of selected product ions for each compound enhanced mass spectrometric selectivity. The product ions of *m/z* 512 and 261 were concluded to be specific for lumefantrine and artesunate.

The LLOQ for lumefantrine was 200 ng/mL. The intra-run precision and intra-run accuracy (% RE) of the LLOQ plasma samples containing lumefantrine were 6.66 and −5.75%, respectively. All the values obtained below 200 ng/mL for lumefantrine were excluded from statistical analysis as they were below the LLOQ values validated for lumefantrine.

#### 3.2.2. Matrix Effect

The assessment of matrix effect constitutes an important and integral part of validation for quantitative LC-MS/MS for supporting pharmacokinetic studies. It was performed by processing six different lots of plasma samples in quadruplet (*n* = 4). LQC and HQC working solutions were spiked after extraction in duplicate for each lot.

**Conclusion 3.2.2 .**

The results found were well within the acceptable limits as the % CV of the area ratios of postspiked recovery samples at LQC and HQC was 4.55% and 5.65%, respectively, which was within 10% for lumefantrine. Hence minor suppression or enhancement of analytes signal due to endogenous matrix interferences did not affect the quantification of lumefantrine.

#### 3.2.3. Linearity, Precision and Accuracy, and Recovery

The peak area ratios of calibration standards were proportional to the concentration of lumefantrine in each assay over the nominal concentration range of 200–20000 ng/mL. The calibration curves appeared linear and were well described by least-squares linear regression lines. 

As compared with the 1/*x* weighing factor, a weighing factor of 1/*x* ^2^ properly achieved the homogeneity of variance and was chosen to achieve homogeneity of variance. The regression squares were greater than 0.9900 for lumefantrine. The deviation of the backcalculated values from the nominal standard concentrations was less than 15%. This validated linearity range justifies the concentration observed during real sample analysis.

The inter-run precision and accuracy were determined by pooling all individual assay results of replicate (*n* = 6) quality control over three separate batch runs analyzed on three different days. The inter-run precision (% CV) and inter-run accuracy (% RE) at LLOQ level were ≤5.56% and ≤−6.02% respectively for lumefantrine. The intra-run precision and accuracy were determined by pooling all individual assay results of replicate (*n* = 6) quality control of two separate batch runs analyzed on the same day. The intra-run precision (% CV) and intra-run accuracy (% RE) at LLOQ level were 6.66% and −5.75%, respectively, for lumefantrine. 

The intra-run and inter-run precision and accuracy data at all quality control level are presented in Table 1. Both the (% CV) precision and accuracy (% RE) at all quality control levels were within 15%, which indicates that the method is precise and accurate.

Six post extracted replicates (samples spiked in extracted matrix of same lot) at low, medium, middle, and high quality control concentration levels for lumefantrine were prepared for recovery determination, and the areas obtained were compared versus the areas obtained for extracted samples (shown in Table 2) of the same concentration levels from a precision and accuracy batch run on the same day.

The mean recovery for lumefantrine was 93.16% with a precision of 3.61% and the mean recovery for artesunate was 91.05% with a precision of 3.80%. This indicates that the recovery for lumefantrine as well as artesunate was consistent and reproducible.

#### 3.2.4. Dilution Integrity and Partial Volume

Dilution integrity and partial volume exercise was performed using six replicates (*n* = 6) of respective samples. The mean back calculated concentrations for 1/2 and 1/4 dilution samples were within 85–115% of their nominal. The % CV for 1/2 and 1/4 dilution samples were 4.46% and 2.35% respectively. The mean backcalculated concentrations for half and quarter partial volume samples were within 85–115% of their nominal. The % CV for half and quarter partial volume samples was 2.18% and 7.17%, respectively. 

#### 3.2.5. Reinjection Reproducibility and Stabilities

Reinjection reproducibility exercise was performed to check whether the instrument performance remains unchanged after hardware deactivation due to any instrument failure during real subject sample analysis. % Change was less than 10.25% for LQC and HQC level concentration; hence batch can be reinjected in case of instrument failure during real subject sample analysis. Also samples prepared were reinjected after 29 hours which shows % change less than 3.46% for LQC and HQC level concentration; hence the batch can be reinjected after 29 hours in case of instrument failure during real subject sample analysis.

Stock solution stability was performed to check stability of lumefantrine and artesunate in stock solutions prepared in methanol: acetonitrile: 0.1 N hydrochloric acid (70 : 30 : 0.05,  v/v/v) and stored at 2–8°C in a refrigerator. The freshly prepared stock solutions were compared with stock solutions prepared before 16 days. The % change for lumefantrine and artesunate was 0.58% and 0.65%, respectively, indicating that stock solutions were stable at least for 16 days.

Bench-top, coolant and autosampler stability for lumefantrine was investigated at LQC and HQC levels. The results revealed that lumefantrine was stable in plasma for at least 17 h at room temperature, 26 h in a coolant at −70°C, and 34 h in an autosampler at 10°C. It was confirmed that repeated freezing and thawing (three cycles) of plasma samples spiked with lumefantrine at LQC and HQC levels did not affect their stability. The long-term stability results also indicated that lumefantrine was stable in matrix up to 221 days at a storage temperature of −70°C. The results obtained from all these stability studies are tabulated in Table 3.

#### 3.2.6. Application

The validated method has been successfully used to quantify lumefantrine concentrations in sixty human volunteers, under fasting conditions after administration of a single tablet containing 120 mg lumefantrine as an oral dose. The study was carried out after approval from an independent ethics committee and after obtaining signed approval from the volunteers.

The pharmacokinetic parameters evaluated were *C*_max_ (maximum observed drug concentration during the study), AUC_0−72_ (area under the plasma concentration-time curve measured 72 hours, using the trapezoidal rule), *T*_max_ (time to observe maximum drug concentration), *K*el (apparent first-order terminal rate constant calculated from a semilog plot of the plasma concentration versus time curve, using the method of least squares regression), and *T*_1/2_ (terminal half-life as determined by quotient 0.693/*K*el).

The mean *C*_max_ that was observed for lumefantrine in case of both test and reference formulations was 4048.66 and 4119.53 ng/mL, respectively. The corresponding mean *T*_max_ that was observed for lumefantrine in case of both test and reference formulations was 1.60 and 1.57 h. The mean AUC_0−72_ that was observed for lumefantrine in case of both test and reference formulations was 26665.94 and 26461.79 ng × h/mL, respectively (data shown in Table 4). The 90% confidence intervals of the ratios of means *C*_max_, AUC_0−72_ all fell within the acceptance range of 80%–125%, demonstrating the bioequivalence of the two formulations of lumefantrine. 

**Figure 4:**
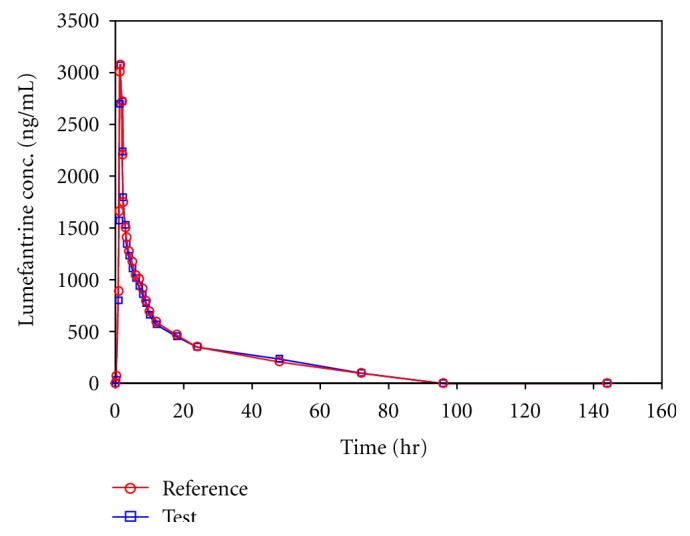
Mean concentration versus time profile of lumefantrine in human plasma from sixty subjects receiving a single oral dose of 120 mg lumefantrine tablet as test and reference.

The mean concentration versus time profile of lumefantrine in human plasma from sixty subjects that are receiving a single oral dose of 120 mg lumefantrine tablet as test and reference is shown in Figure 4.

## 4. Conclusion

The developed LC-MS/MS assay for lumefantrine is rapid, selective, and suitable for routine measurement of subject samples. 

The present method provided excellent specificity and linearity with a limit of quantification of 200 ng/mL for lumefantrine, which is sufficient enough to give data for calculation of the required pharmacokinetic data and establish bioequivalence. The other major advantage of this validated method is the run time of 2.5 minutes which allows the quantitation of over 150 plasma samples per day.

## Figures and Tables

**Table 1:** Intrarun and interrun precision and accuracy (*n* = 6) of lumefantrine in human plasma.

Run	Concentration added (ng/mL)	Mean concentration found (ng/mL)	% CV	% RE
Intra-	201.01	189.45	6.66	−5.75
	600.70	560.22	4.95	−6.74
	10011.60	9566.39	4 .88	−4.45
	17019.71	15705.69	3.69	−7.72
	20023.19	18693.52	6.11	−6.64

Inter-	201.01	188.90	5.56	−6.02
	600.70	581.70	5.99	−3.16
	10011.60	9727.42	9.14	−2.84
	17019.71	16097.79	4.87	−5.42
	20023.19	18804.76	3.80	−6.09

CV: coefficient of variation; RE: relative error.

**Table 2:** Recovery for lumefantrine and artesunate (*n* = 6).

Analytes	Level	*A*	B	% Recovery	% CV
Lumefantrine	LQC	17238	15995	92.85	4.51
	MQC	271999	251471	92.47	3.05
	HQC	440485	414535	94.15	3.55

Artesunate	LQC	226450	200282	88.50	2.90
	MQC	214968	196401	91.37	1.55
	HQC	210154	195829	93.27	4.59

*A*: mean area of unextracted sample (*n* = 6); *B*: mean area of extracted sample (*n* = 6); mean recovery was found to be 93.16% for lumefantrine and 91.05 for artesunate; CV: coefficient of variation.

**Table 3:** Stability results for lumefantrine (*n* = 6).

Stability	Level	*A*	% CV	*B*	% CV	% Change
Autosampler (34 h, 10°C)	LQC	633.33	3.38	605.19	4.77	−4.44
	HQC	15829.36	1.40	15232.73	1.93	−3.77
Bench top (17 h at room temp.)	LQC	633.33	3.38	600.27	1.51	−5.22
	HQC	15829.37	1.40	15292.07	3.51	−3.38
Coolant (26 h, −70°C)	LQC	633.33	3.38	622.32	3.91	−1.74
	HQC	15829.37	1.40	15514.03	1.58	−1.99
Reinjection (29 h, 2–8°C)	LQC	555.86	4.90	575.11	5.28	3.46
	HQC	16044.53	2.95	15570.59	2.52	−2.95
3rd freeze-thaw cycle (−70°C)	LQC	578.49	6.01	581.93	9.99	−0.59
	HQC	14834.03	6.68	16427.64	4.79	10.74
Long term (221 days, −70°C)	LQC	555.86	4.90	578.54	3.67	4.08
	HQC	16044.53	2.95	17745.02	3.86	10.60

*A*: mean value of comparison samples (original concentrations before storage) concentrations (ng/mL); *B*: mean value of stability samples (measured concentration after storage) concentrations (ng/mL); CV: coefficient of variation; h: hours, temp: temperature.

**Table 4:** Pharmacokinetic parameters of lumefantrine using non-compartmental analyses.

Parameters	Lumefantrine	
	Mean ± SD	
	Test	Reference
*C*_max_ (ng/mL)	4048.66 ± 511.45	4119.53 ± 475.21
AUC_0–72_ (ng × h/mL)	26665.94 ± 9199.99	26461.79 ± 8578.98
*T*_max_ (h)	1.60 ± 0.34	1.57 ± 0.32
*K*_el_ (h^−1^)	0.03 ± 0.02	0.03 ± 0.02
*T*_1/2_ (h)	40.05 ± 23.72	34.78 ± 23.63
